# Choriocapillaris Flow Impairments in Association with Pachyvessel in Early Stages of Pachychoroid

**DOI:** 10.1038/s41598-019-42052-w

**Published:** 2019-04-03

**Authors:** Jiwon Baek, Lee Kook, Won Ki Lee

**Affiliations:** 1 Department of Ophthalmolog, Bucheon St. Mary’s Hospital, College of Medicine, The Catholic University of Korea, Gyeoggi-do, South Korea; 2 Department of Ophthalmology, Seoul St. Mary’s Hospital, College of Medicine, The Catholic University of Korea, Seoul, South Korea

## Abstract

To analyze features of the choriocapillaris in eyes with earlier stages of pachychoroid spectrum, this study included 46 eyes with PPE, 32 age-matched eyes with pachychoroid without epitheliopathy, and 30 normal controls. Macular 3 × 3 mm^2^ angiographic images were obtained with swept-source optical coherence tomography. Vascular density and signal void area in the choriocapillaris were analyzed. Topographical correlation of signal voids with the dilated choroidal large vessel (pachyvessel) was assessed. Choriocapillaris vascular density was significantly lower in eyes with PPE compared with controls (*p* = 0.003). The number, the total area and the average size of signal voids was the highest in the PPE group followed by the pachychoroid without epitheliopathy and then controls (all *p* ≤ 0.001). 89.0% signal void area colocalized with pachyvessels. The average size of the signal void was higher if it was colocalized with pachyvessel (*p* < 0.001). In conclusion, the area of flow impairment in the choriocapillaris was increased in eyes with pachychoroid and even greater when epitheliopathy was present. Pachyvessel was associated with choriocapillaris flow impairment by location and size.

## Introduction

Developments in imaging technology have enabled visualization of the choroid by introducing enhanced depth imaging (EDI) mode to optical coherence tomography (OCT). Margolis and Spaide^1^ broadened the use of this technology to assessment of choroidal morphology, and the term ‘pachychoroid’ was coined several years ago by Warrow *et al*.^2^ Pachychoroid is now commonly used to describe a specific morphology of the choroid found in a spectrum of diseases as well as quantitative choroidal thickening. The concept of pachychoroid has changed the paradigm of macular diseases including central serous chorioretinopathy (CSC) and age-related macular degeneration (AMD).

While the morphological features of pachychoroid are clearly defined, the pathogenesis of pachychoroid still requires further elucidation. Most studies indicate that AMD starts in Bruch’s membrane and the retinal pigment epithelium^3–5^. The change in choroid is considered as a secondary event rather than a primary event. However, in pachychoroid-associated diseases, the pathognomonic finding that defines the condition lies within the choroid. Dansingani *et al*.^6^ revealed that focal choroidal thickening in pachychoroid could be attributed to pathologically dilated Haller vessels (pachyvessels), which lead to overlying inner choroid and retinal pigment epithelium (RPE) thinning, and these alterations in the choroidal vascular structure were specified qualitatively and quantitatively in pachychoroid spectrum disease in subsequent studies^7,8^. The results from these studies led to the hypothesis that changes in the choroid may play a major part in the pathogenesis in pachychoroid diseases.

One of the suggested mechanisms for choroidal involvement in the pathogenesis of pachychoroid disease is that the ischemic milieu to the RPE can be produced by choriocapillaris attenuation which might be evident from collapsed inner choroidal layer on OCT b-scans. We speculated that quantitative analysis of choriocapillaris flow impairment using the method described by Spaide^9^ in eyes with early pachychoroid spectrum stages can address whether choriocapillaris flow impairment is a preceding condition for further changes in RPE and retina. Therefore, this study was performed to quantitatively analyze flow features of the choriocapillaris in eyes with early pachychoroid spectrum stages, including pachychoroid eyes without retinal changes and pachychoroid pigment epitheliopathy (PPE) eyes. We also examined the topographical correlation with underlying pachyvessels using OCT angiography (OCTA).

## Results

### Baseline characteristics

A total of 108 eyes from 108 patients were included in this study. The study samples included 46 PPE, 32 Pachy, and 30 control eyes. All patients were Korean. The mean age was 54.2 ± 9.7 years (range, 32–74 years), and 71 (66%) patients were male. The mean age, gender distribution, laterality of the study eye, BCVA, and refractive errors did not differ among the PPE, Pachy, and control groups (*p* = 0.142, 0.642, 0.151, 0.224, and 0.206, respectively). There was no significant difference for these parameters when compared between each group. The mean subfoveal choroidal thickness of the PPE and Pachy groups was thicker compared to controls (both *p* < 0.001), while there was no difference between the PPE and Pachy groups (*p* = 0.137). Prevalence of choroidal caverns was higher in PPE and Pachy groups compared to controls (*p* = 0.007 and 0.010, respectively). Patient baseline characteristics are summarized in Table 1.

**Table 1:** Baseline characteristics of the patients.

Factors	All eyes (n = 108)	PPE (n = 46)	Pachychoroid (n = 32)	Controls (n = 30)	*p*-value^a^	*p*-value^b^	*p*-value^c^	*p*-value^d^
Age (years), Mean ± SD (Range)	54.2 ± 9.7 (32–74)	55.5 ± 10.5 (32–70)	55.1 ± 10.4 (32–74)	51.3 ± 6.8 (42–68)	0.142	0.870	0.053	0.092
Gender, male/female	63/45	32/14	19/13	20/10	0.642	0.352	0.790	0.553
Laterality, OD/OS	52/56	27/19	12/20	13/17	0.151	0.066	0.190	0.469
BCVA (LogMAR), Mean ± SD (Range)	0.05 ± 0.09 (0–0.40)	0.06 ± 0.01 (0–0.40)	0.04 ± 0.06 (0–0.20)	0.04 ± 0.06 (0–0.20)	0.224	0.194	0.154	0.710
RE (diopters), Mean ± SD (Range)	−0.45 ± 1.43 (−4.50 – + 4.25)	−0.44 ± 1.47 (−4.50 – +4.25)	−0.15 ± 1.18 (−4.25 – +1.75)	−0.80 ± 1.57 (−4.50 – +1.00)	0.206	0.346	0.320	0.074
Choroidal thickness (um), Mean ± SD (Range)	366.1 ± 100.6 (191–632)	381.0 ± 101.0 (223–632)	411.0 ± 75.2 (303–585)	295.4 ± 87.7 (191–551)	<0.001*	0.137	<0.001*	<0.001*
Presence of choroidal caverns, n (%)	16 (16)	9 (20)	7 (22)	0 (0)	0.026*	0.804	0.010*	0.007*

PPE: pachychoroid epitheliopathy; SD: standard deviation; OD: OD: oculus dexter; OS: oculus sinister; BCVA: best-corrected visual acuity; RE: refractive error;

*Statistically significant p-value.

^a^ANOVA or chi-square test between all groups.

^b^Independent t-test or chi-square test between pachychoroid and PPE.

^c^Independet t-test or chi-square test between control and PPE.

^d^Independent t-test or chi-square test between controls and pachychoroid.

### Flow features of choriocapillaris

The mean vascular density of choriocapillaris was lowest in the PPE group and highest in controls (*p* = 0.048). Vascular density was significantly lower in PPE compared to Pachy group and controls (*p* = 0.003 and 0.044, respectively). The mean number of signal voids per eye was highest in the PPE group and lowest in controls (ANOVA; *p* < 0.001, LSD; both *p* < 0.001). The mean total area of signal voids per eye was also greatest in the PPE group and least in controls (ANOVA; *p* < 0.001, LSD; PPE vs. Pachy *p* < 0.001, PPE vs. control *p* < 0.001, and Pachy vs. control *p* < 0.001). The average area per signal void was greater in PPE eyes compared to Pachy group and controls (*p* = 0.004 and <0.001, respectively). The average area per signal void in the Pachy group was greater compared to controls (*p* = 0.029). The standard deviation (SD) of signal void area per eye was also higher in PPE eyes compared with Pachy and control eyes (*p* = 0.002 and <0.001, respectively), and it was higher in Pachy group than controls (*p* = 0.010). Choricapillaris features are summarized in Table 2.

**Table 2:** Flow features of choriocapillaris.

Parameters	All eyes	PPE	Pachychoroid	Controls	*p* -value^a^	*p* -value^b^	*p* -value^c^	*p* -value^d^
	(n = 108)	(n = 46)	(n = 32)	(n = 30)				
Vascular density (%) Mean ± SD (Range)	66.50 ± 0.02 (51.4–69.5)	65.97 ± 1.22 (62.6–67.7)	66.75 ± 1.02 (65.2–58.6)	67.03 ± 3.21 (51.4–69.5)	0.048*	0.003*	0.044*	0.648
Number of singnal voids (n) Mean ± SD (Range)	400.1 ± 98.0 (167–706)	462.7 ± 90.1 (304–706)	390.0 ± 58.7 (277–640)	315.0 ± 72.9 (167–436)	<0.001*	<0.001*	<0.001*	<0.001*
Total area of singnal voids (mm^2^) Mean ± SD (Range)	0.0478 ± 0.0277 (0.010–0.147)	0.0644 ± 0.0306 (0.0291–0.1475)	0.0419 ± 0.0138 (0.028–0.097)	0.0287 ± 0.0117 (0.002–0.0693)	<0.001*	<0.001*	<0.001*	<0.001*
Average area of singnal void (μm^2^) Mean ± SD (Range)	115.1 ± 44.0 (45–344)	136.5 ± 52.9 (84–344)	106.6 ± 23.9 (72–190)	91.5 ± 28.7 (445–168)	<0.001*	0.004*	<0.001*	0.029*
SD of singnal voids (μm^2^) Mean ± SD (Range)	39.6 ± 33.4 (8–242)	56.0 ± 44.6 (17–242)	31.1 ± 11.6 (15–62)	23.4 ± 11.0 (8–54)	<0.001*	0.003*	<0.001*	0.010*

PPE: pachychoroid epitheliopathy; SD: standard deviation *Statistically significant *p*-value. ^a^ANOVA test between all groups. ^b^independent t-test between pachychoroid and PPE. ^c^Independet t-test between control and PPE. ^d^independent t-test between controls and pachychoroids.

### Topographical correlation between choriocapillaris signal voids and pachyvessels

In total, 72.5% of the number of signal voids (31339/43212 voids) and 89.0% of the area of signal voids (4.60 mm^2^/5.16 mm^2^) were located over pachyvessels. The proportions of number and area of signal voids that co-localized with pachyvessels in PPE, Pachy, and control groups were 73.9% and 90.9%, 75.0% and 85.8%, and 66.2% and 82.7%, respectively. The topographical correlation was strongest in the PPE group and weakest in controls (ANOVA *p* < 0.001 for both number and area of signal voids). The average area per signal void on pachyvessels was greatest in the PPE group (both *p* < 0.001 compared with Pachy and controls) and least in controls, but the difference was not significant between Pachy and control groups (*p* = 0.325). The SD of signal void area per eye was higher in PPE eyes compared with Pachy and controls (*p* = 0.002 and <0.001), but no significant difference was found between the Pachy group and controls (*p* = 0.056). In the signal voids that colocalized with pachyvessels, the average size of the signal void was larger compared with those that were not located on pachyvessels (140.4 ± 57.3 μm^2^, range, 50.7–437.9 μm^2^ vs. 48.4 ± 27.8 μm^2^, range, 17.5–525.02, *p* < 0.001). Only 2/14 (14%) choroidal caverns were colocalized with the signal void area. Topographical associations between signal voids and pachyvessel are summarized in Table 3.  The dataset for this study is provided as supplementary file.

**Table 3:** Flow features of choriocapillaris in association with pachyvessel.

Parameters	All eyes	PPE	Pachychoroid	Controls	*p* -value^a^	*p* - value^b^	*p* - value^c^	*p* - value^d^
	(n = 108)	(n = 46)	(n = 32)	(n = 30)				
N. of singnal voids on pachyvessel (n) Mean ± SD (Range)	290.2 ± 85.1 (111–573)	342.5 ± 77.2 (227–573)	292.4 ± 55.8 (173–527)	207.6 ± 52.3(111–296)	<0.001*	0.001*	<0.001*	<0.001*
Proportion to total voids (%) Mean ± SD (Range)	72.07 ± 8.30 (41.7–97.6)	73.93 ± 6.59 (62.6–97.6)	74.96 ± 8.09 (62.5–96.4)	66.15 ± 8.12 (41.7–86.8)	<0.001*	0.553	<0.001*	<0.001*
Total singnal voids area (mm^2^) Mean ± SD (Range)	0.0426 ± 0.0263 (0.007–0.143)	0.0592 ± 0.033 (0.026–0.143)	0.0360 ± 0.0126 (0.023–0.093)	0.0241 ± 0.0111 (0.007–0.064)	<0.001*	<0.001*	<0.001*	<0.001*
Proportion to total voids (%) Mean ± SD (Range)	87.11 ± 7.12 (40.1–99.3)	90.92 ± 4.68 (78.0–99.3)	85.81 ± 7.10 (40.1–96.6)	82.66 ± 7.35 (66.5–95.1)	<0.001*	<0.001*	<0.001*	0.092
Average area of singnal void (μm^2^) Mean ± SD (Range)	140.4 ± 57.3 (51–437)	169.4 ± 69.5 (91–437)	122.9 ± 30.6 (76–177)	114.5 ± 35.4 (51–233)	<0.001*	<0.001*	<0.001*	0.325
SD of singnal voids Mean ± SD (Range)	42.5 ± 31.6 (8–194)	58.2 ± 40.4 (20–194)	34.3 ± 13.3 (17–76)	27.1 ± 15.7 (8–73)	<0.001*	0.002*	<0.001*	0.056

PPE: pachychoroid epitheliopathy; SD: standard deviation. *Statistically significant *p*-value. ^a^ANOVA test between all groups. ^b^Independent t-test between pachychoroid and PPE. ^c^Independet t-test between control and PPE. ^d^Independent t-test between controls and pachychoroid.

## Discussion

In eyes with pachychoroid spectrum diseases, obvious changes in the choroid including enlargement of the vascular space in Haller’s layer and the compressed choriocapillaris layer were present. These changes are almost always spotted under the disease foci and some other parts of the choroid of the same eye^6,7^. Although the change in choroid might be focal in some cases (especially in polypoidal choroidal vasculopathy eyes with thin/subnormal choroid)^8^, most of the eyes with thickened subfoveal choroid showed global dilatation of Haller vessels^10,11^. This global change in Haller vessels seems to be intrinsic rather than secondary to retinal changes^12–14^, which could serve as an underlying pathogenic mechanism for pachychoroid disorders. Therefore, detailed information on the anatomy and functional features of choroidal vasculature can help further our understanding on the disease pathogenesis. In the current study, we compared choriocapillaris flow features in terms of signal voids and vascular density using OCTA. One strength of this study is that it is a comparative study in which earlier stages of pachychoroid spectrum diseases were included. Furthermore, this study quantitatively analyzed topographical correlations between pachyvessel and signal void area.

Our results showed that the number and area of choriocapillaris signal voids were greater in eyes with pachychoroid compared to controls and even greater when epitheliopathy was present. These observations have two important implications. First, more prominent choriocapillaris signal void number and area in PPE suggests that choriocapillaris flow impairment may get worse as epitheliopathy progresses, or the progression of signal voids may result in epitheliopathy. Second, the higher number and greater area of signal voids in pachychoroid eyes before epitheliopathy (the Pachy group) compared to controls suggests that choriocapillaris flow impairment can exist without RPE or outer retinal change. This means that choriocapillaris flow impairment may precede RPE or outer retinal change in pachychoroid eyes, and choroidal vascular change may be a causative event for further changes.

In the current study, the average size of the choriocapillaris signal void was higher in the PPE group compared with the Pachy and control groups. Gal-Or *et al*.^15^ reported a fairly high prevalence of relatively large flow signal attenuation zones in eyes with chronic CSC and PPE. Although the method for measurement of flow attenuation was different between our study and the previous report, these results suggest that larger void areas (or larger signal attenuation zones) are more likely to be pathologic. The higher SD of signal voids together with higher mean and total area of signal voids in PPE compared to both Pachy group and controls can be interpreted in this regard. Higher variation in the size with larger area of signal voids in PPE eyes is attributable to the presence of larger attenuation zones in these eyes.

In the analysis of the topographical correlation between signal voids and pachyvessels, 89.0% of the signal void area was co-localized with pachyvessels. The spatial correlation of choriocapillaris flow and pachyvessel was addressed in recent studies^15–17^. Gal-Or *et al*.^15^ identified zones of reduced choriocapillaris flow that were anatomically correlated with pachyvessels and structural sequelae in the RPE and retina in eyes with chronic CSC and PPE. Matet *et al*.^16^ demonstrated that choriocapillaris signal voids were colocalized with choriocapillaris thinning and Haller vessel dilation in CSC eyes by manual measurement of choriocapillaris upon deep choroidal vessel layer OCT b-scan. Yun *et al*.^17^ showed colocalization of choriocapillaris flow and the choroid vascular bed just below the choriocapillaris (which they obtained from 60–117 μm below the Bruch’s membrane). In the current study, choriocapillaris flow disturbances were automatically measured as signal voids and their topographical relationship with pachyvessel was assessed using *en face* OCTA. The method used in this study can substantiate quantitative topographical correlations between choriocapillaris signal voids and pachyvessels in a more direct manner compared with previous studies. Additionally, the size of signal voids was greater if it was colocalized with pachyvessels. These results, along with findings of previous studies, suggest that the development of choriocapillaris flow impairment may be associated with pachyvessels. The detailed underlying mechanism requires further investigation.

The number and area of signal voids was higher in the Pachy group compared to controls. Signal voids also showed a high topographical correlation with pachyvessels (75% by number and 85% by area). Based on this finding, we speculate that the increase in choriocapillaris flow impairment that colocalizes with pachyvessels exists in pachychoroid eyes even before the appearance of pathologic changes in the RPE or outer retina. However, the average size of signal voids in the Pachy group was smaller compared to PPE eyes. Here, the process of the disease can be inferred to be a fundamental generalized impairment of choriocapillaris flow followed by enlargement of the flow impairment area.

The prevalence of choroidal caverns was relatively low compared to a previous study reported choroidal caverns in pachychoroid eyes (52%)^18^. The difference can be explained with inclusion of eyes at different stages of disease spectrum between these studies. The subjects included in the previous study include eyes with later stages in pachychoroid diseases (i.e. PPE, CSC, and pachychoroid neovasculopathy), while this study only includes eyes at earlier stages. This hypothesis also has an implication that choroidal cavern may be associated with disease progression, but further studies are warranted for this. Additionally, limited area of 3 × 3 mm^2^ imaging field of OCTA would have resulted in the lower prevalence of choroidal caverns in this study. Although the topographical correlation between the choroidal caverns and choriocapillaris signal voids, this also requires further study with larger numbers of caverns, especially in eyes with later stages of pachychoroid.

This study has limitations inherent to its hospital-based and retrospective study design. There might be selection bias, and the comparison of other imaging modalities to detect choroidal vessel status, such as fluorescein angiography or indocyanine green angiography, was not possible. In addition, due to resolution limits in the current OCTA system, this study only analyzed signal void area for the purpose of studying the anatomical structure of the choriocapillaris. Whether the signal voids we analyzed are actually areas absent of vascular components or flow requires further verification. Nonetheless, since the objects included in the study were PPE or eyes with normal retinas, the interference in image analysis caused by abnormal retinal structures such as fibrovascular membrane, fluid accumulation, hemorrhage, or exudates has been minimized.

In summary, choriocapillaris flow impairments exist in eyes with pachychoroid and their location was correlated with the location of pachyvessels. With the finding that choriocapillaris flow impairment existed before any epitheliopathy in eyes with pachychoroid, these results suggest that choriocapillaris flow change may be a preceding event before further development of retinal disease, such as PPE, CSC, or eventually neovascularization. Further studies regarding the mechanism by which pachyvessels can cause choriocapillaris flow impairment, or vice versa, are warranted.

## Methods

This study was performed at the Department of Ophthalmology in Seoul St. Mary’s Hospital at The Catholic University of Korea. This study was approved by the Institutional Review Board of the Seoul St. Mary’s Hospital College of Medicine, which waived the written informed consent because of the study’s retrospective design and was conducted in accordance with the tenets of the Declaration of Helsinki.

We recruited consecutive patients with PPE (PPE group), patients with pachychoroid eye without changes in the retina (Pachy group), and patients with normal choroid and retina (controls) that visited our hospital between June 2017 and March 2018. Diagnosis of pachychoroid was based on previously established definitions^2,19^, (i.e. subfoveal choroidal thickness >300 μm or an extrafoveal focus that exceeded fovea choroidal thickness by at least 50 μm), and the presence of pachyvessels identified on OCT b-scans and *en face* images^6^. Pachyvessels were defined as dilated outer choroidal vessels extending into the macula observed on SS-OCT *en face* slabs of outer choroid, which correlated with the areas of maximal choroidal thickness with increased Haller layer proportion. PPE diagnosis was based on previous definitions^2,19^. Findings of epitheliopathy for the definition of PPE included pachydrusen with or without pigmentary changes on color fundus photograph and focal loss or thinning of the interdigitation zone, ellipsoid zone, and external limiting membrane with or without focal thinning of the outer nuclear layer on OCT b-scans. Age-matched normal controls were recruited from patients presenting with floaters. Medical records were reviewed to access patient clinical history and imaging data.

Exclusion criteria were as follows: (a) any history of previous treatments that could cause significant changes to the choroid, such as laser photocoagulation and intraocular, periocular, or systemic corticosteroids; (b) other concomitant eye conditions including high myopia (>−6 diopter or axial length >26 mm), retinal detachment, macular hole, uveitis, diabetic retinopathy, or glaucoma; and (c) severe media opacity that could degrade image quality such as cataracts and vitreous opacity.

All patients underwent a comprehensive ocular examination that included Snellen best-corrected visual acuity (BCVA), auto-refractometry (KR-1, Topcon Corporation, Tokyo, Japan), color fundus photography (DRI OCT Triton, Topcon Corporation), and swept source OCT (SS-OCT; DRI OCT Triton) line + volume scan and OCTA scan using SS-OCT.

### Imaging Analysis

Total choroidal thickness, defined as the distance between the Bruch membrane and the choroid-scleral border, was measured using OCT horizontal b-scans intersecting the center^8^. On horizontal raster b-scans that correspond 3 × 3 mm^2^ OCTA area, choroidal caverns were identified using the definition previously described, as gaping angular hyporeflective cavities^18,20^.

**Figure 1:**
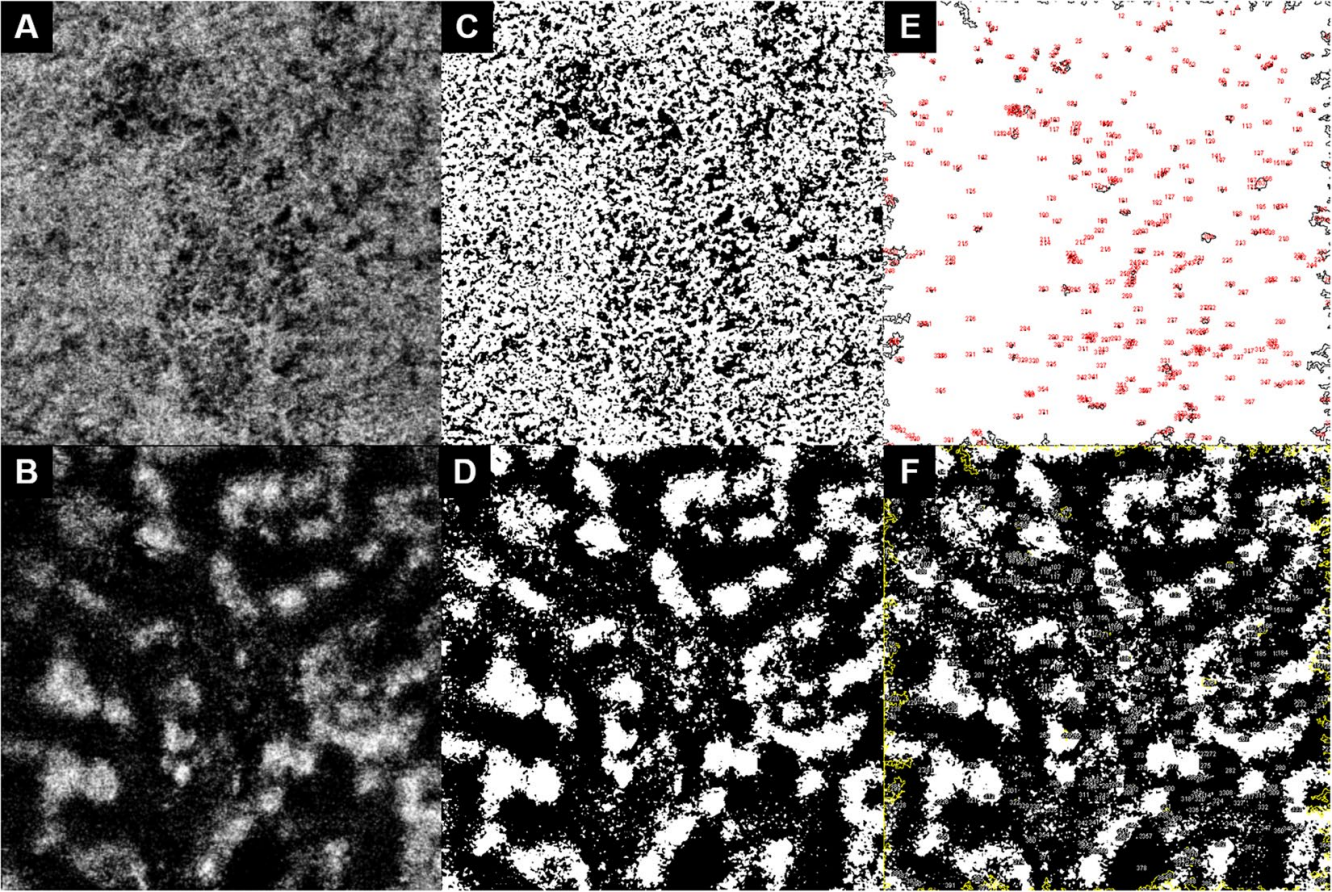
Analysis of optical coherence angiographic images. Macular 3 × 3 mm^2^ angiographic images obtained from the right eye of 68 year-old male with pachychoroid pigment epitheliopathy. *En face* OCTA images of the macular area centered at the fovea from the choriocapillaris layer (from the Bruch’s membrane to 10.4 μm thickness of the inner choroid, (**A**) and Haller’s layer (by moving the reference line to the point of 50% of the total choroidal thickness, (**B**) were obtained. Binarization of the two slabs was performed using automatic local thresholding with the Phansalkar method for the choriocapillaris slab (**C**) and Niblack method for the Haller vessel slab (**D**). Applying the “Analyze Particles” to the binarized choriocapillaris slab, the total number and area of signal voids were obtained (**E**). The average size per signal void was calculated as total area divided by number of signal voids. Signal voids were selected as region of interests (ROIs) and the selection was overlaid onto the binarized Haller vessel slab for the assessment of topographical correlation of signal voids and pachyvessel (**F**). Total number and area of signal voids and the mean area per signal void were also obtained for signal voids that colocalized with pachyvessels.

*En face* 3 × 3 mm^2^ OCTA images of the macular area centered at the fovea from the choriocapillaris layer and Haller’s layer were obtained. A choriocapillaris slab was obtained by automatic segmentation using ImageNet software (ImageNet 6, version 1.19.11030, Topcon Corporation), which shows flow structures from the Bruch’s membrane to 10.4 μm thickness of the inner choroid (Fig. 1A). A Haller vessel slab was obtained by moving the reference line to a point that was 50% of the total choroidal thickness (Fig. 1B). First, binarization of the two slabs was performed using FIJI software (an expanded version of ImageJ version 1.51a, available at fiji.sc, free of charge). Automatic local thresholding using the Phansalkar method with a radius of 15 pixels was applied for the choriocapillaris slab^9^ and Niblack binarization with a radius of 30 pixels was applied for the Haller vessel slab^10^ (Fig. 1C,D). The vascular density was calculated by dividing the number of pixels in the vascular area (dark pixels) by that of the total area of both slabs.

The binarized choriocapillaris slab was then analyzed with the “Analyze Particles” command, which measured and counted all thresholded areas greater than or equal to one pixel where there was a lack of flow information (signal void)^9^. The total number and area of signal voids and the mean area per signal void were obtained (Fig. 1E). Signal voids were selected as region of interests (ROIs) and the selection was overlaid onto the binarized Haller vessel slab (Fig. 1F). The pixel gray intensity scale for the ROIs (pure black 0 to pure white 255) on the Haller vessel slab was measured for the topographical correlation of signal voids and pachyvessels. The signal void ROIs measured as black were considered colocalized with pachyvessels. Total number and area of signal voids and the mean area per signal void were also obtained for signal voids that colocalized with pachyvessels.

### Statistical analysis

Statistical analysis was performed with SPSS for Windows (version 23.0.1; SPSS Inc., Chicago, IL, USA). Independent t-tests and one-way analysis of variance (ANOVA) were used to compare continuous variables among and between groups. Mann-Whitney and Kruskal-Wallis tests were used when a normal distribution could not be confirmed. Paired t-tests were used to compare signal void areas according to their correlation with pachyvessels. Fisher’s least significant difference (LSD) was used as the post-hoc test after ANOVA. Categorical variables between groups were compared using the chi-square test. Standardized adjustment was used as the post-hoc test after the chi-square test. A *p* value < 0.05 was considered statistically significant.

## Supplementary information

Supplementary Dataset 1

## Data Availability

The datasets generated during and/or analysed during the current study are available from the corresponding author on reasonable request.
